# Comparison of Machine Learning Computed Tomography-Based Fractional Flow Reserve and Coronary CT Angiography-Derived Plaque Characteristics with Invasive Resting Full-Cycle Ratio

**DOI:** 10.3390/jcm9030714

**Published:** 2020-03-06

**Authors:** Stefan Baumann, Markus Hirt, Christina Rott, Gökce H. Özdemir, Christian Tesche, Tobias Becher, Christel Weiss, Svetlana Hetjens, Ibrahim Akin, Stefan O. Schoenberg, Martin Borggrefe, Sonja Janssen, Daniel Overhoff, Dirk Lossnitzer

**Affiliations:** 1 First Department of Medicine-Cardiology, University Medical Centre Mannheim, and DZHK (German Centre for Cardiovascular Research), partner site Heidelberg/Mannheim, Mannheim 68167, Germany; markus.hirt@stud.uni-heidelberg.de (M.H.); tina.rott@gmx.de (C.R.); goekce-oezdemir@msn.com (G.H.Ö.); tbecher@mail.rockefeller.edu (T.B.); ibrahim.akin@umm.de (I.A.); martin.borggrefe@umm.de (M.B.); dirk.lossnitzer@umm.de (D.L.); 2 European Center for Angioscience, Mannheim 68167, Germany; 3 Department of Internal Medicine, St. Johannes-Hospital, Dortmund 44137, Germany; tesche.christian@gmail.com; 4 Laboratory of Molecular Metabolism, The Rockefeller University, New York NY 10065, USA; 5 Medical Faculty Mannheim, Department of Medical Statistics and Biomathematics, University Medical Center Mannheim, Heidelberg University, Mannheim 68167, Germany; Christel.Weiss@medma.uni-heidelberg.de (C.W.); Svetlana.Hetjens@medma.uni-heidelberg.de (S.H.); 6 Institute of Clinical Radiology and Nuclear Medicine, University Medical Center Mannheim, Faculty of Medicine Mannheim, Heidelberg University, Mannheim 68167, Germany; stefan.schoenberg@umm.de (S.O.S.); Sonja.Janssen@umm.de (S.J.); Daniel.Overhoff@umm.de (D.O.)

**Keywords:** atherosclerosis, coronary artery disease, coronary CT angiography, fractional flow reserve derived from coronary computed tomography angiography, coronary physiology, invasive coronary angiography, myocardial infarction, resting full-cycle ratio, revascularization

## Abstract

Background: The aim is to compare the machine learning-based coronary-computed tomography fractional flow reserve (CT-FFR_ML_) and coronary-computed tomographic morphological plaque characteristics with the resting full-cycle ratio (RFR^TM^) as a novel invasive resting pressure-wire index for detecting hemodynamically significant coronary artery stenosis. Methods: In our single center study, patients with coronary artery disease (CAD) who had a clinically indicated coronary computed tomography angiography (cCTA) and subsequent invasive coronary angiography (ICA) with pressure wire-measurement were included. On-site prototype CT-FFR_ML_ software and on-site CT-plaque software were used to calculate the hemodynamic relevance of coronary stenosis. Results: We enrolled 33 patients (70% male, mean age 68 ± 12 years). On a per-lesion basis, the area under the receiver operating characteristic curve (AUC) of CT-FFR_ML_ (0.90) was higher than the AUCs of the morphological plaque characteristics length/minimal luminal diameter^4^ (LL/MLD^4^; 0.80), minimal luminal diameter (MLD; 0.77), remodeling index (RI; 0.76), degree of luminal diameter stenosis (0.75), and minimal luminal area (MLA; 0.75). Conclusion: CT-FFR_ML_ and morphological plaque characteristics show a significant correlation to detected hemodynamically significant coronary stenosis. Whole CT-FFR_ML_ had the best discriminatory power, using RFR^TM^ as the reference standard.

## 1. Introduction

Assessment of coronary artery stenosis with coronary computed tomographic angiography (cCTA) and additional computed tomographic morphological plaque characteristics derived from cCTA datasets leads to a more detailed evaluation of suspicious stenosis [1]. Two studies published recently showed that the joined assessment of some computed tomographic morphological plaque characteristics and cCTA could improve the detection of hemodynamically relevant stenosis [2,3]. In addition, the functional relevance of a lesion may be assessed by cCTA-based fractional flow reserve (CT-FFR) [4]. Several studies have shown a significant correlation between non-invasive CT-FFR and invasive fractional flow reserve (FFR) [2,5,6]. Using an on-site prototype with a machine-learning algorithm (CT-FFR_ML_) to reduce computation time was the consequent next step in technical development [7,8,9,10]. CT-FFR_ML_ was compared in previously published studies to the invasive gold standards invasive FFR [8] and instantaneous wave-free ratio (iwFR) [11] and showed promising results.

Invasive diagnostic tools currently used to assess the hemodynamic relevance of coronary artery stenosis during invasive coronary angiography (ICA) are invasive FFR and iwFR [12,13]. These two methods are both recommended by the 2018 European Society of Cardiology (ESC) guidelines on myocardial revascularization as a class IA recommendation [12,14,15].

Resting full-cycle ratio (RFR^TM^) is a recently developed invasive parameter to determine the hemodynamic relevance of coronary artery stenosis. Compared to invasive FFR, it is not necessary to induce hyperemia for RFR^TM^, resulting in improved patient comfort. The advantage of this novel resting index approach may consist of detecting lower values during systole, while iwFR measures values during diastole. RFR^TM^ showed an excellent correlation to the gold standard iwFR in recent studies [16,17].

The aim of our study is to compare the machine learning-based coronary-computed tomography fractional flow reserve (CT-FFR_ML_) and coronary-computed tomographic morphological plaque characteristics calculated based on coronary-computed tomography angiography (cCTA) with the resting full-Cycle ratio (RFR^TM^).

## 2. Materials and Methods

### 2.1. Study Design and Patient Population

The local Institutional Review Board approved the study protocol (No. 2015-583N-MA). Written informed consent was obtained from all patients. Patients with suspected coronary artery disease (CAD) underwent a clinically indicated cCTA and ICA with pressure wire measurement. The CAD consortium clinical score was applied to determine the pre-test probability for CAD and estimate the probability based on age, sex, symptoms, and cardiovascular risk factors [18]. The acquired cCTA datasets were used to calculate CT-FFR_ML_ and computed tomographic morphological plaque characteristics. Four patients had to be excluded due to inadequate image quality. Patients were included if they had at least one coronary artery with stenosis of undetermined hemodynamic relevance, defined as a 40% to 70% diameter stenosis by visual assessment [15]. These lesions were further assessed by pressure wire measurements (Verrata^TM^ pressure wire, Volcano Corporation, Koninklijke Philips N.V. Amsterdam, The Netherlands) [14]. After the procedures, the raw data of the iwFR measurement were extracted and analyzed with the RFR^TM^ algorithm by an external core lab (Abbott GmbH & Co. KG; Wiesbaden, Germany). Exclusion criteria for cCTA were electrocardiographic signs of acute myocardial ischemia, unstable angina with elevated serum cardiac biomarkers, patients with renal insufficiency, and known contrast agent allergies. The exclusion criteria for CT-FFR_ML_ calculation and computed tomographic plaque characteristics analysis included left main or complex bifurcation stenosis, aneurysms, severe diffuse disease, chronic total occlusion, previous percutaneous coronary stent implantation or coronary artery bypass graft (CABG), or inadequate image quality. Data for baseline characteristics were obtained from electronic medical records.

### 2.2. Acquisition and Analysis of cCTA Datasets

A third-generation dual-source CT (Siemens Somatom FORCE, Siemens Healthineers, Forchheim, Germany) was used for imaging. A specified regime of medication consisting of sublingual nitroglycerin (0.8 mg) and intravenous beta-blockers were given prior to the scan if deemed necessary by a radiologist. Initially, 80 mL iodinated contrast material (Iomeron 400; Bracco Imaging S.p.A., Milan, Italy) was administered using a power injector (Stellant D; Medrad, Warrendale, PA, USA) at a flow rate of 5 mL/s followed by a 50 mL saline chaser. Coronary artery stenosis was analyzed and quantified using on-site software (Coronary Plaque Analysis 2.0. syngo. via FRONTIER, Siemens Healthineers). The grading was done in accordance with the guidelines of the Society of Cardiovascular Computed Tomography [19].

### 2.3. Analysis of Machine Learning Computed Tomography-Based Fractional Flow Reserve and Computed Tomographic Morphological Plaque Characteristics

The Leiden-risk score that combines different plaque characteristics to optimize risk stratification was calculated to characterize the complexity and severity of CAD [20]. To quantify coronary calcification, the Agatston score was calculated using a software application, according to the Agatston scoring convention (CaScore, Siemens Healthineers) [21]. The cCTA datasets were used for morphological plaque analysis calculated by on-site software (Coronary Plaque Analysis 2.0.). The target lesion was defined as an area with atherosclerotic changes between non-affected proximal and distal sections without any atherosclerotic changes. Discrepancies in plaque density measured in Hounsfield units (HU) were used to analyze the plaque morphology [2]. The software automatically calculated the parameters lesion length (LL), vessel volume (VV), and total plaque volume (TPV) [3]. LL was defined as the dimension of the plaque between areas free of atherosclerotic plaque. Minimal luminal area (MLA) was measured automatically by the on-site prototype software as the lowest value for the luminal area within the lesion. Corrected coronary opacification (CCO) was defined as the difference between the lowest mean HU attenuation at the proximal extent of stenosis and the lowest mean HU attenuation in the coronary artery distal to the lesion and was calculated manually [22]. The minimal luminal diameter (MLD) was measured, although manually, as the area with the narrowest luminal diameter in the lesion [2]. The remodeling index (RI) was calculated as the ratio of the smallest cross-sectional area of the atherosclerotic lesion over the respective proximal luminal area [23]. On-site CT-FFR prototype software based on a machine-learning algorithm (Siemens cFFR, version 3.1; Siemens Healthineers, currently not commercially available) was used and installed on a regular workstation (Syngo VE36A; Siemens Healthineers) for the CT-FFR_ML_ calculation. An experienced cardiovascular radiologist checked the semi-automatically calculated vessel-specific centerlines for the accurate recognition of suspected coronary artery stenosis. The exact percentage of manual centerline corrections was initially not documented. However, the algorithm has captured excellently in most cases, and the rate of manual corrections was fewer than 5%. The on-site prototype software calculated blood flow and showed the hyperemic state in the coronary vessels based on patient-specific physiological conditions and flow dynamic models. The technical specification of the used CT-FFR_ML_ algorithm is described in detail in a previously published study [8]. After on-site calculation, the CT-FFR_ML_ software created a patient-specific anatomic color-coded 3-dimensional mesh of the coronary artery tree and aortic root. A CT-FFR_ML_ cut off value of ≤ 0.80 was used for the detection of hemodynamically relevant coronary artery stenosis [24].

### 2.4. Invasive Coronary Angiography and Resting Full Cycle Ratio Measurement

A coronary-pressure guide wire (Verrata^TM^ pressure wire, Volcano Corporation) was introduced in the respective coronary artery [12,15]. The pressure sensor was placed distal to the stenosis. The optimal diastolic interval with minimized and constant microvascular resistance for pressure measurement was calculated by dedicated software (Volcano Corporation). IwFR values ≤ 0.89 were considered as diagnostic relevant for lesion-specific ischemia [12,15,25]. The raw data of the iwFR measurement was extracted from the console and an external core lab (Abbott GmbH & Co. KG) retrospectively calculated the RFR^TM^ with the new algorithm, using a hyperemia-free resting measurement for the coronary pressure at the lowest point of resting diastolic-pressure-to-aortic-pressure ratio. The maximal relative pressure difference in the whole cardiac cycle (either in systole or diastole) in five consecutive cardiac cycles was measured. The cut-off value for hemodynamic relevance for RFR^TM^ was equal to the value for iwFR ≤ 0.89 [16,17].

### 2.5. Statistical Analysis

SAS (version 9.4, SAS Institute Inc., Cary, NC, USA) was used for all analyses. For quantitative variables, mean values and standard deviations were calculated. Categorical variables are presented as percentages, whereas continuous variables are presented as either mean ± standard deviation (SD) or median with interquartile range (IQR) and were analyzed with the independent sample *t*-test. For qualitative factors, absolute and relative frequencies are presented. In order to quantify the degree to which two measurement methods agree, interclass correlation (ICC) was estimated using a two-way ANOVA with one of the methods being equivalent to 100% consistency. Additionally, Kappa coefficients were calculated to assess the degree of agreement for binary factors. Sensitivity, specificity, positive predictive value, and negative predictive value were evaluated on a per-lesion and per-patient level for CT-FFR_ML_ and coronary-computed tomographic morphological plaque characteristics calculated based cCTA, using RFR^TM^ (≤0.89) as the reference standard to detect lesion-specific ischemia. The area under the receiver operating characteristics curve (AUC) was determined and compared for cCTA, CT-FFR_ML_, and RFR^TM^ as a metric of overall diagnostic performance. For computed tomographic morphological plaque characteristics that showed statistically significant correlation with the RFR^TM^ values, sensitivity, specificity, positive predictive value, and negative predictive value were calculated. A *p*-value of ≤0.05 was considered statistically significant. The AUC was determined for the significant CT-FFR_ML_ and morphological plaque characteristics as a metric of overall diagnostic performance.

## 3. Results

We enrolled 33 patients (70% male, mean age 68 ± 12 years) with acquired cCTA datasets, CT-FFR_ML_ and morphological plaque characteristics calculations, and RFR^TM^ measurements. On average, we calculated a pretest probability of 57% ± 19%, which was calculated with the CAD consortium clinical score [18]. Baseline characteristics of patients are presented in Table 1.

**Figure 1:**
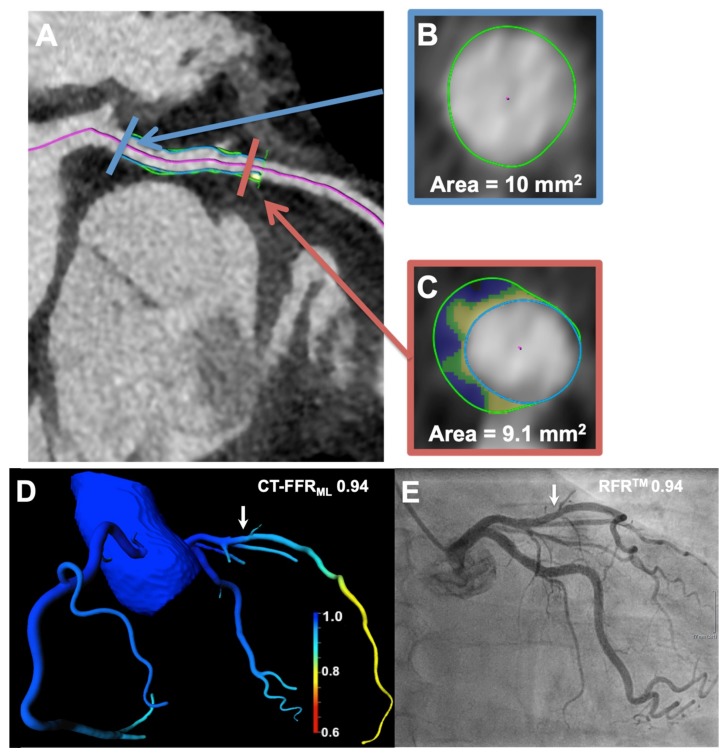
(**A**) The acquired cCTA shows non-relevant stenosis of the LAD with color-coded automated lesion quantification by the plaque tool. The mixed plaque in the LAD demonstrated a remodeling index of 0.91 and was calculated as (**C**) the target-lesion cross-sectional area (marked in orange). (**B**) divided by proximal reference-cross-sectional area (marked blue). (**D**) A 3-dimensional color-coded reconstruction calculated CT-FFR_ML_ value of 0.94 (CT-FFR_ML_ cut-off value ≤ 0.80) is presented (arrow). (**E**) This stenosis (arrow) was visualized by ICA and was invasively measured using RFR^TM^ (0.94; RFR^TM^ cut-off value ≤ 0.89), indicating no hemodynamic relevance. cCTA = coronary computed tomography angiography; CT-FFR_ML_ = fractional flow reserve derived from coronary computed tomography angiography based on machine learning algorithm; ICA = invasive coronary angiography; LAD = left anterior descending artery; RFR^TM^ = resting full-cycle ratio.

Forty-four vessel lesions in 33 patients were analyzed. Fourteen (32%) were identified as hemodynamically relevant stenoses by RFR^TM^ (RFR^TM^ ≤ 0.89), whereas CT-FFR_ML_ classified only 13 (30%) as hemodynamically significant coronary artery stenoses (CT-FFR_ML_ ≤ 0.80). The mean calculation time for the CT-FFR_ML_ was 11 ± 2 min, and the mean calculation time for the analysis of the morphological plaque markers was 15 ± 5 min. An example case is presented in Figure 1.

The cCTA-derived quantitative markers, LL/MLD^4^, MLD, the degree of luminal diameter stenosis, MLA, CT-FFR_ML_, and RI, showed statistically significant differences between obstructive and non-obstructive lesions compared to the reference standard RFR^TM^ (Table 2).

Lesion length (LL), total plaque volume (TPV), vessel volume (VV), and corrected coronary opacification (CCO) did not achieve statistical significance and were unable to detect lesion-specific ischemia (Table 2). We further assessed the sensitivity, specificity, positive predictive values and negative predictive values and accuracy for CT-FFR_ML_ and morphological plaque characteristics with statistical significance as well as the area under the receiver operating characteristic curve (AUC).

On a per-lesion and per-patient level, for CT-FFR_ML_, the parameter with the highest discriminatory power, the sensitivity, specificity, positive predictive value, and a negative predictive value are demonstrated in Table 3.

Accordingly, LL/MLD^4^, as the best representative coronary computed tomographic morphological plaque characteristic, on a per-lesion and per-patient basis yielded a sensitivity of 71% (95% CI: 42–92%) and 75% (95% CI: 73–94%), a specificity of 77% (95% CI: 58–90%) and 71% (95% CI: 48–84%), a positive predictive value of 59% (95% CI: 33–82%) and 60% (95% CI: 32–84%), and a negative predictive value of 85% (95% CI: 66–96%) and 83% (95% CI: 59–96%), respectively. In comparison, using RFR^TM^ as the reference standard, the sensitivity, specificity, positive predict value, negative predict value and accuracy for MLD, the degree of luminal diameter stenosis, MLA, RI, cCTA (>50%), and cCTA (>70%) are lower than CT-FFR_ML_ and LL/MLD^4^ (Table 3) for detecting lesion-specific ischemia.

When compared to RFR^TM^, the diagnostic accuracy for detecting hemodynamically significant coronary artery stenoses of CT-FFR_ML_ and LL/MLD^4^ on a per-lesion and a per-patient level was 93% (95% CI: 81–98%) and 91% (95% CI: 76–98%) and 75% (95% CI: 60–87%) and 73% (95% CI: 55–87%), respectively. For MLD, the degrees of accuracy of luminal diameter stenosis, MLA, RI, cCTA (>50%), and cCTA (>70%) are also listed in Table 3.

The ROC curve analysis on a per lesion level for CT-FFR_ML_ and coronary-computed tomographic morphological plaque characteristics resulted in an AUC of 0.90 for CT-FFR_ML_, 0.80 for LL/MLD^4^, 0.77 for MLD, 0.75 for the degree of luminal diameter stenosis, 0.77 for MLA, 0.76 for RI, 0.69 for cCTA (<50%), and likewise, 0.69 for cCTA (<70%). The AUCs on a per-patient level are outlined in Table 3.

## 4. Discussion

In this study, the aim is to compare CT-FFR_ML_ and coronary computed tomographic morphological plaque characteristics calculated with the RFR^TM^ as a novel invasive resting pressure-derived index for detecting hemodynamically significant coronary artery stenosis.

Based on the comparatively moderate specificity of cCTA, a tendency may exist to overestimate the severity of coronary artery stenosis by cCTA examination [26,27,28]. In order to compensate for this disadvantage of cCTA, which is caused by its low specificity for the detection of hemodynamically relevant stenosis, techniques have been developed in recent years to improve the quantification of suspicious coronary artery stenosis.

The coronary computed tomographic morphological plaque characteristics calculated based on cCTA were able to prove in various studies and the information gained from plaque parameters led to an improvement in the diagnostic accuracy of the cCTA for the detection of hemodynamically relevant stenosis [2,3,22]. Our results demonstrate that cCTA morphological plaque characteristics, including LL/MLD^4^, MLD, the degree of luminal diameter stenosis, MLA, and RI, have discriminatory power to differentiate between hemodynamically relevant and non-relevant coronary artery stenoses. In line with previous studies, LL/MLD^4^, MLD, the degree of luminal diameter stenosis, and MLA were also identified as having significant discriminatory power in our study [3,22]. The present analysis supports findings from Tesche et al. [3], Wang et al. [22], and Baumann et al. [29], that LL, TPV, and VV do not show statistically relevant differences between flow obstructing and non-flow obstructing coronary artery stenoses. However, our findings concerning the discriminatory power of RI and CCO are discordant with the results of the studies mentioned above regarding cCTA plaque characteristics. We could demonstrate that the RI belongs to the morphological plaque characteristics with significant discriminatory power. Previous studies have described the opposite results when examining the discriminatory power of RI [3,22]. Even when using CCO, our results differed from the studies mentioned. We could further demonstrate that CCO did not show statistically significant differences between relevant and non-relevant coronary artery lesions. In contrast to these studies mentioned above, with the exception of one study, which used the invasive FFR as the reference standard, we utilized the RFR^TM^ as a novel invasive resting index and to detect hemodynamically significant coronary artery stenosis. We could demonstrate that LL/MLD^4^, an anatomic plaque marker, had the best diagnostic power to determine the hemodynamic relevance of coronary lesions on a per-lesion level (AUC: 0.80; 95% CI: 0.64–0.96%). Our results are in accordance with the findings of a previous study, including Baumann et al. [29] (AUC: 0.84) and Wang et al. [22] (AUC: 0.90) that also demonstrated the utility of LL/MLD^4^. We are also able to support the findings in terms of MLD (AUC: 0.81 and AUC: 0.80). In addition to our previously published results [29], it can be assumed that some plaque markers derived from regular anatomical cCTA may assist in the detection of hemodynamically relevant coronary stenosis. Specifically, LL/MLD^4^, a morphological plaque marker, with its high specificity and accuracy, may resemble a strong predictor.

In addition to morphological plaque characteristics calculated by cCTA, suspicious coronary lesions may be further assessed by functional cCTA predictors. As an example, CT-FFR is gaining importance for non-invasive assessment of suspicious coronary artery stenosis. Previous trials, including DISCOVER-FLOW, DeFACTO, and NXT, have demonstrated a good correlation between CT-FFR and invasive FFR [24,30,31]. These results led to the approval of the CT-FFR off-site algorithm by the US Food and Drug Administration (FDA) in 2015.

Because of the time-consuming calculation time by the off-site CT-FFR algorithm, an on-site CT-FFR_ML_ algorithm was developed [7]. This on-site CT-FFR_ML_ algorithm shortened calculation time and has been successfully validated since [10]. In our study, we utilized this on-site CT-FFR_ML_ algorithm, which is currently not commercially available. In addition, both off-site and on-site CT-FFR algorithms were compared to invasive gold standard FFR in the above-mentioned studies. We could demonstrate similar results in terms of diagnostic accuracy (accuracy on a per-lesion level: 93%, 95% CI: 81–98%) and a high diagnostic performance for the CT-FFR_ML_ (AUC: 0.90, 95% CI: 0.75–1.00%) using the RFR^TM^ as a novel invasive resting pressure-derived index for detecting hemodynamically significant coronary artery stenosis.

Instead of using the invasive gold standard FFR, the novel resting pressure-derived index RFR^TM^ was used as the reference standard to classify suspicious coronary artery stenosis. Initial studies have demonstrated that both CT-FFR_ML_ and morphological plaque characteristics compare well to iwFR as the reference standard for detecting hemodynamically significant coronary artery stenosis regarding their diagnostic accuracy [11,29,32]. Thus, we used the rest index RFR^TM^ as the reference standard, as RFR^TM^ demonstrated an excellent correlation to iwFR [16]. Therefore, this is the first study that compares CT-FFR_ML_ and morphological plaque characteristics based on cCTA with the RFR^TM^ as a novel invasive resting pressure-derived index for detecting hemodynamically significant coronary artery stenosis.

Our findings are to be evaluated and considered in light of the following limitations. First, it should be noted that our monocentric study cohort of a total number of 33 enrolled patients with 44 lesions is relatively small. Second, we did not perform any comparisons to conventional invasive FFR as the invasive gold standard.

Third, all patients had suspected but chronic coronary syndrome with an indication for cCTA, which is just a subgroup of patients who could possibly have an advantage of improved diagnostic tools. We treated patients according to existing guidelines, stating that invasive pressure wire measuring is only indicated in patients lacking evidence of ischemia. Finally, there are no follow up data regarding any adverse events or survival rates stratified by diagnostic parameter or treatment.

## 5. Conclusions

In contrast to prior studies using invasive FFR or iwFR as the reference standard, this is the first investigation to our knowledge to compare CT-FFR_ML_ and morphological plaque characteristics against the invasive reference standard RFR^TM^ as a novel resting index without the use of intra-arterial adenosine administration. On-site CT-FFR_ML_ and computed tomography morphological plaque characteristics (LL/MLD^4^, RI, MLD, MLA, degree of luminal diameter stenosis) showed a significant correlation to detected hemodynamically significant coronary artery stenosis. Whole CT-FFR_ML_ had the best discriminatory power using RFR^TM^ as the reference standard. CCO, LL, TPV, and VV failed to detect hemodynamically relevant coronary artery stenosis.

## Figures and Tables

**Table 1:** Baseline characteristics (patients *n* = 33 and lesions ^π^ *n* = 44) and findings of cCTA, CT-FFR_ML_, and ICA.

Parameter	Mean Value ± Standard Deviation, Frequency (%) or Interquartile Range
Age (years)	68 ± 12
Men	23 (70%)
Body mass index (kg/m^2^)	29 ± 5
Pretest probability (%) ^+^	57 ± 19
**Cardiovascular Risk Factors**	
Hypertension *	26 (78%)
Hyperlipidemia ^†^	18 (54%)
Diabetes mellitus	8 (24%)
Family history of coronary artery disease	8 (24%)
Current smoker	4 (12%)
**Coronary Computed Tomography**	
Agatston score	800 (35–3608)
Luminal stenosis > 70%	14 (32%)
Leiden cCTA risk score	12 ± 5
CT-FFR_ML_ ≤ 0.80 ^π^	13 (30%)
**Invasive Coronary Catheter Angiography**	
Left anterior descending coronary artery ^π^	26 (59%)
Left circumflex coronary artery ^π^	10 (23%)
Right coronary artery ^π^	8 (18%)
RFR^TM^ ≤ 0.89 ^π^	14 (32%)

Unless otherwise specified, data are numbers of patients with percentages in parentheses. Data are mean ± standard deviation (SD) or interquartile range. ^+^ Pretest probability calculated with the CAD consortium clinical score [18]. * Defined as blood pressure > 140 mmHg systolic, >90 mmHg diastolic, or use of an antihypertensive medication. ^†^ Defined as a total cholesterol level of >200 mg/dL or use of antilipidemic medication; cCTA = coronary computed tomography angiography; CT-FFR_ML_ = fractional flow reserve derived from coronary computed tomography angiography based on machine learning algorithm; RFR^TM^ = resting full-cycle ratio.

**Table 2:** Comparison of CT-FFR_ML_ and plaque characteristics in stenosis with and without hemodynamic relevance stenosis using RFR^TM^ as reference.

Parameter	All Lesions	Lesions RFR^TM^ > 0.89	LesionsRFR^TM^ ≤ 0.89	*p*-Value
Number of Lesions	44	30	14	-
**Morphological computed tomographic morphological plaque characteristics**				
LL/MLD^4^	11.0 ± 6.2	8.8 ± 4.0	15.7 ± 7.6	0.0016 *
MLD (mm)	1.8 ± 0.8	2.0 ± 0.8	1.4 ± 0.7	0.0038 *
Degree of luminal diameter stenosis (%)	50.3 ± 28.6	42.9 ± 26.3	66.4 ± 27.4	0.0063 *
MLA (mm^2^)	4.8 ± 3.5	5.5 ± 3.4	3.5 ± 3.3	0.0078 *
cCTA stenosis > 50%	26	14	12	0.0141 *
cCTA stenosis > 70%	14	6	8	0.0341 *
TPV (mm^3^)	116.1 ± 76.2	101.4 ± 66.3	147.5 ± 88.5	0.0989
VV (mm^3^)	200.7 ± 117.0	189.0 ± 115.7	225.7 ± 120.0	0.3710
LL (mm)	17.0 ± 7.4	16.5 ± 8.1	18.2 ± 5.7	0.4421
**Functional computed tomographic morphological plaque characteristics**				
CT-FFR_ML_	0.87 ± 0.14	0.94 ± 0.05	0.72 ± 0.15	<0.0001 **
RI	1.20 ± 0.28	1.12 ± 0.23	1.38 ± 0.29	0.0062 *
CCO	0.13 ± 0.12	0.14 ± 0.13	0.13 ± 0.08	0.6770

cCTA = coronary computed tomography angiography; CCO = corrected coronary opacification; CT-FFR_ML_ = fractional flow reserve derived from coronary computed tomography angiography based on machine learning algorithm; LL = lesion length; MLA = minimal luminal area; MLD = minimal luminal diameter; RFR^TM^ = resting full-cycle ratio; RI = remodeling index; TPV = total plaque volume; VV = vessel volume; ** highly significant (*p* ≤ 0.001), * significant (*p* ≤ 0.05).

**Table 3:** Diagnostic performance of fractional flow reserve derived from coronary-computed tomography angiography based on machine learning algorithm and coronary-computed tomographic plaque characteristic on a per-lesion and per-patient level using RFR^TM^ as the reference standard.

Per Lesion (*n* = 44)						
	Sensitivity (%) (95% CI)	Specificity (%) (95% CI)	PPV (%) (95% CI)	NPV (%) (95% CI)	Accuracy (%) (95% CI)	AUC (95% CI)
**Morphological Computed Tomographic Morphological Plaque Characteristics**						
LL/MLD^4^	71 (42–92)	77 (58–90)	59 (33–82)	85 (66–96)	75 (60–87)	0.80 (0.64–0.96)
MLD (mm^2^)	64 (35–87)	80 (61–92)	60 (32–84)	83 (64–94)	75 (59–87)	0.77 (0.62–0.93)
Degree of luminal diameter stenosis (%)	71 (42–92)	77 (58–90)	59 (33–82)	85 (66–96)	75 (59–87)	0.75 (0.58–0.94)
MLA (mm^2^)	71 (42–92)	80 (61–92)	62 (35–85)	86 (67–96)	77 (62–88)	0.75 (0.57–0.94)
cCTA (<50%)	86 (57–98)	53 (34–72)	46 (27–67)	89 (65–98)	63 (48–78)	0.69 (0.56–0.83)
cCTA (<70%)	57 (29–82)	80 (61–92)	57 (29–82)	80 (61–92)	73 (57–85)	0.69 (0.53–0.84)
**Functional Computed Tomographic Morphological Plaque Characteristics**						
CT-FFR_ML_	86 (57–98)	97 (88–99)	92 (74–99)	94 (79–99)	93 (81–98)	0.90 (0.75–1.00)
RI	71 (42–92)	67 (47–83)	50 (27–73)	83 (63–95)	68 (52–81)	0.76 (0.61–0.91)
**Per Patient (*n* = 33)**						
**Morphological Computed Tomographic Morphological Plaque Characteristics**						
LL/MLD^4^	75 (73–94)	71 (48–84)	60 (32–84)	83 (59–96)	73 (55–87)	0.78 (0.57–0.99)
MLA (mm^2^)	67 (35–90)	71 (48–89)	57 (29–82)	79 (54–94)	70 (51–84)	0.70 (0.48–0.93)
MLD (mm^2^)	58 (28–85)	71 (48–89)	54 (25–81)	75 (51–91)	67 (48–82)	0.70 (0.50–0.90)
Degree of luminal diameter stenosis (%)	67 (35–93)	67 (43–85)	53 (27–79)	78 (52–94)	67 (48–82)	0.68 (0.46–0.89)
cCTA (<50%)	83 (52–98)	62 (39–82)	44 (23–65)	80 (44–98)	55 (36–72)	0.60 (0.45–0.76)
cCTA (<70%)	50 (21–79)	71 (48–89)	50 (21–79)	71 (48–88)	64 (45–80)	0.60 (0.42–0.78)
**Functional Computed Tomographic Morphological Plaque Characteristics**						
CT-FFR_ML_	83 (52–98)	95 (76–99)	91 (59–99)	91 (71–99)	91 (76–98)	0.87 (0.70–1.00)
RI	67 (35–90)	52 (30–74)	44 (21–69)	73 (45–92)	58 (39–74)	0.70 (0.51–0.89)

AUC = area under the receiver operating characteristic curve; cCTA = coronary computed tomography angiography; CI= confidence interval; CT-FFR_ML_ = fractional flow reserve derived from coronary computed tomography angiography based on machine learning algorithm; MLA = minimal luminal area; MLD = minimal luminal diameter; NPV = negative predictive value; PPV = positive predictive value; RFR^TM^ = resting full-cycle ratio; RI = remodeling index.
